# Medicinal Plant Use in North Karelia, Finland, in the 2010s

**DOI:** 10.3390/plants14020226

**Published:** 2025-01-15

**Authors:** Renata Sõukand, Natalia Kuznetsova, Julia Prakofjewa, Sabira Ståhlberg, Ingvar Svanberg, Baiba Prūse, Giulia Mattalia, Raivo Kalle

**Affiliations:** 1 Department of Environmental Sciences, Informatics & Statistics, Ca’ Foscari University of Venice, 30173 Venice, Italy; 2 Department of Linguistic Sciences and Foreign Literatures, Catholic University of the Sacred Heart, 20123 Milan, Italy; 3 Institute for Russian and Eurasian Studies, Uppsala University, SE-75120 Uppsala, Sweden; 4 MaREI, The Research Ireland Centre for Energy, Climate, and Marine, Environmental Research Institute, University College Cork, P43 C573 Cork, Ireland; 5 Institute of Environmental Science and Technology, Autonomous University of Barcelona (ICTA-UAB), 08193 Barcelona, Spain; 6 Estonian Literary Museum, 51003 Tartu, Estonia

**Keywords:** North Karelia, ethnomedicine, borderland, plant-based remedies, herbals, written sources, herbalists, cultural transmission, healthcare systems, historical ethnobotany

## Abstract

Finnish North Karelia is a region with a rich cultural history of ethnomedicinal plant use, shaped by centuries of interactions among various ethnic groups. This study identified both similarities and divergences between local Finns, Karelians war refugees, and individuals of mixed origin compared to historical records. Based on 67 semi-structured interviews, we documented the use of 43 medicinal plant taxa from 25 families, of which 31 remain in use. Notably, the number of medicinal plants continuously used in North Karelia is considerably lower than in other parts of Europe, with less than 25% of historically utilised species still in practice, which reflects the fragile state of this knowledge. Factors such as forced relocation, the loss of traditional lands, and the need to adapt to new environments might have contributed to this decline. Another influencing factor is official healthcare attitudes, which have prompted Finnish residents to shift from traditional herbal remedies to modern medical practices. Understanding the circulation of ethnomedicinal knowledge and its transformation over time is essential for identifying pathways to revitalise these practices within the framework of modern healthcare systems and cultural revitalisation efforts.

## 1. Introduction

The use of plants is guided by various historical, socio-political, cultural, linguistic, and religious factors. When multiple cultures intersect, minority groups often adopt components of dominant cultures in their use of plants (e.g., [1,2]), integrating these components into their practices but often also keeping the specific cultural markers that help them to stand out and sustain their identity. This integration or the maintenance of one’s own tradition extends to various aspects of knowledge, including indigenous and local plant knowledge. Indeed, various studies demonstrate that the exchange of local plant knowledge frequently manifests different degrees of cultural adaptations in favouring the practices of dominant groups [3,4,5].

Similarly, the homogenisation of local plant knowledge, particularly in the medicinal domain, has been guided by herbalists through medical instruction for centuries [6,7]. These processes intensified during the Enlightenment, especially in the 20th century with globalisation and the development of such centralised national health systems that contained medicinal plants in routine circulation [8,9].

Consequently, being embedded into new environments and cultures, the richness of indigenous and local plant knowledge undergoes profound transformations. This change affects not only the abundance of local plant knowledge of minority groups but also the mechanisms through which this knowledge is passed on to successive generations [10].

Diversity, by definition, inherently resists homogenisation. Regions rich in linguistic diversity often coincide with areas of high biodiversity, as evidenced by the cooccurrence of linguistic and biological diversity in many parts of the world [11]. However, the effect of culturally diverse and/or multilingual backgrounds on indigenous and local ecological knowledge corpora has yet to be studied. It could be supposed that those with diverse inputs can choose between different sources of knowledge and therefore possess a broader spectrum compared to those who have grown up in one dominant culture (although all cultures and languages are, to a certain extent, the result of the mixtures of different cultures and languages).

Prehistoric cultivation and gathering practices in Finland have been widely examined to understand the historical relationship between plants and humans [12]. During the medieval period, monastic communities became centres of horticultural expertise, cultivating medicinal herbs for both therapeutic and spiritual purposes. The first nuns and monks of the Naantali Cloister (Southwest Finland), sent from Vadstena Abbey in Sweden, likely introduced medicinal garden plants and gardening skills to the region [13].

The ethnobotanical practices of Finland have been documented in numerous ethnographic and historical accounts [14,15,16]. Limited but valuable details on the use of plants in the Northern Karelian part of Finland were documented by Manninen [17]. A significant focus has been on Sámi communities in northern Fennoscandia, where studies have documented the integration of indigenous plant knowledge with modern scientific understanding [18,19]. Despite their rich ecological knowledge and deep connection to the Arctic environment, the Sámi face significant vulnerabilities that threaten their traditional way of life in modern settings [20,21].

While the ethnobotanical knowledge of specific groups like the Sámi and historical practices in Finland have been studied, there is limited research on the interplay between multiethnic and multilingual influences and the transmission of medicinal plant knowledge. Furthermore, little is known about how individuals exposed to multiple cultural contexts navigate and retain ethnomedicinal practices, especially in regions like Finnish Karelia, where diverse cultural contexts intersect. This gap leaves questions about the influence of cultural diversity on the preservation, adaptation, and application of plant knowledge in contemporary and historical contexts.

In this article, we chose Finnish North Karelia, a multicultural region in Northern Europe, to develop the idea that exposure to and engagement with two or more cultures since childhood may affect the knowledge of medicinal plants. This is what should happen in the absence of solid control over local medicinal knowledge. Unlike regions with strong national homogenisation policies, the Finnish state did not actively promote the medicinal use of plants between the 1950s and 1980s, thereby limiting centralisation’s effects [9]. Some historical resources [22] allow for a comparison of recent practices with historical plant uses. Additionally, Finnish historical ethnomedicine has been significantly influenced by written sources, owing to the high literacy rate of the population as early as the beginning of the 20th century [23,24,25]. Therefore, we:

- documented the ethnomedicinal use of plants among Karelians (and their descendants, including those of Karelians war refugees and mixed origin) and local Finns residing in or originating from Finnish North Karelia.
- identified similarities and divergences between the studied groups in their medicinal plants and uses.
- recognised similarities and divergences between the current ethnomedicinal practices and those reported in historical literature.

In the discussion, we contextualise our findings by comparing them with studies from neighbouring regions influenced by state-regulated, centralised medicinal plant systems. We hypothesise that North Karelia will exhibit a smaller repertoire of plants used but a broader diversity of applications, as well as more diverse ethnomedicinal knowledge among individuals raised by parents from distinct cultural backgrounds.

## 2. Results

We documented the current and past medicinal uses of 43 plant taxa (five of them identified at the genus level) belonging to 25 plant families (Table 1).

Only 18 medicinal plant taxa were mentioned by three or more people, while the total number of use reports (DUR) was 322. The most frequently named families were Ericaceae (70 DUR), Pinaceae (60 DUR), Grossulariaceae (49 DUR), Plantaginaceae (32 DUR), and Rosaceae (25 DUR). The most popular taxa were *Ribes nigrum* (45 DUR), *Pinus sylvestris* (37 DUR), *Vaccinium myrtillus* (36 DUR), *Plantago major* (32 UR), *Picea abies* (23 DUR), *Vaccinium oxycoccos* (18 DUR), *Betula* (15 DUR), *Nicotiana rustica* (13 DUR), and *Vaccinium vitis-idaea* (10 DUR). The most widespread uses were for treating skin conditions (81 DUR), the respiratory system (74 DUR), general or unspecified diseases (69 DUR), and digestive tract problems (36 DUR). The use of specific taxa for the general disease category overlapped for at least three people in 21 taxon–use combinations. The general Informant Consensus Factor (ICF) was 0.85, with the ICF for the disease categories being relatively low, highest for ear diseases (0.83), skin (0.82), urological diseases (0.73), and respiratory diseases (0.72), while general and unidentified diseases and digestive diseases had values of 0.67 and 0.52, respectively.

Nine interviewees (three from each ethnic group) did not report using any medicinal plants. Hence, the number of interviewees (n) in Table 2 is smaller than the overall total. The birth year influenced only the mixed Karelian-Finnish (Mix) group, being highest for those born around the 1960s (55–70 years old), although the mean age did have a significant influence (*p* = 0.027) and the Mix group in general had a higher mean of used plants (*p* = 0.026). Additionally, women were more knowledgeable of medicinal plants and their uses (*p* < 0.001) than men.

**Figure 1:**
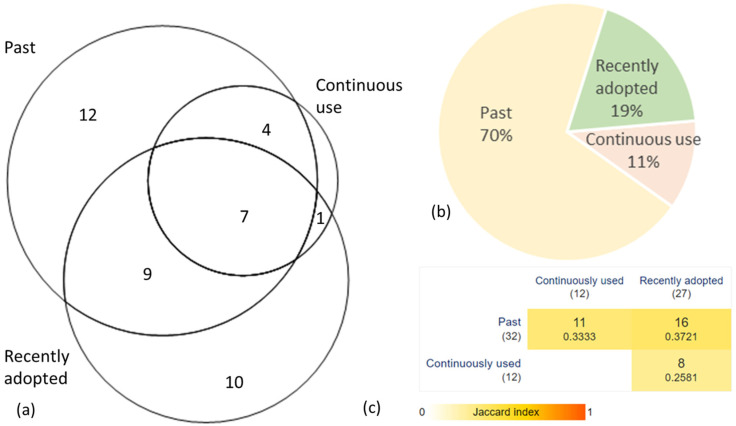
Knowledge circulation in time. (**a**) Number of taxa with different plant use periods. (**b**) Proportion of DURs in different use periods. (**c**) The Jaccard Index signals little overlap between the species used in different periods.

Only 12 taxa have been used continuously (Figure 1a,c), while most currently used plants have been recently adopted. Based on the number of plant taxa, past uses are more numerous than the recently adopted ones, and at the level of use records, past uses dominate (70%) (Figure 1b).

While the majority of disease categories show continuity in knowledge circulation, three disease categories are present in only one of the periods. The “ear” disease category has remained in the past, while the use for men’s and women’s diseases is currently adopted. Notably, the emic category “healthy” (meaning a plant generally good for health) was mentioned 19 times, sharing second and third place in frequency with “flu”, following “wounds”, the most mentioned category.

A total of 98 DUR (30%) were reported for personal use and based on personal knowledge, while 208 DUR (65%) were reported as being used within the family based on family tradition. The remaining 16 DUR (5%) belonged to the prescriptions by a village traditional healer from Lieksa, Anni Pölönen (1889–1960), also known as *Viekin mummo* [Viekki’s Grandma]. Either the plant itself or a medicine made of it by Pölönen was prescribed. For example, *Daphne mezereum* was used against toothache and rickets. The healer followed in the footsteps of her father and grandfather, both of whom were healers.

**Figure 2:**
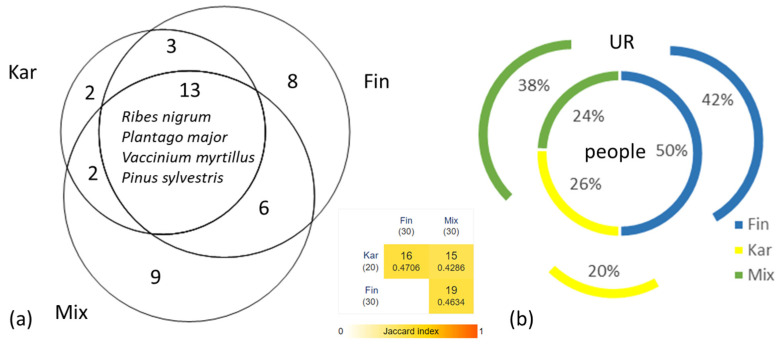
Overlap in the ethnomedicinal knowledge of plants: (**a**) Cross-cultural comparison of all used plants. The four listed species were reported by at least 50% of the interviewees. (**b**) Correspondence between the percentage of people in the group and the percentage of the knowledge of the use of the plants they shared. The Jaccard Index indicates a relatively high diversity between the studied groups.

Four plants used by at least 50% of the interviewees were shared amongst all three groups (Figure 2a). Although the mixed group was the smallest, it had a proportionally highest shared of knowledge (Figure 2b).

The plant names listed by the interviewees were primarily in Finnish. Among the medicinal plants mentioned, only a few (forest) berries (*Rubus*, *Vaccinium*, and *Ribes*); one tree (*Pinus sylvestris*); and one weed (*Urtica dioica*) were named in Karelian (Table 1).

**Figure 3:**
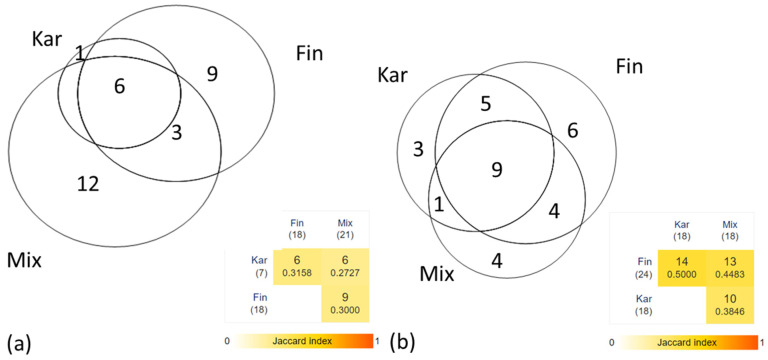
Comparison between the numbers of the used plants (**a**) currently and (**b**) in the past between different groups of respondents (Kar, Fin, and Mix). The past use is more homogenous compared to the current use of plants. The Jaccard Index indicates highly diverse current use, while the past remembered use is more homogenous.

While the plants used only in the past were reported homogeneously (Figure 3b), the currently used plants are rather group-specific (except for Karelians, who used only one plant (*Matricaria chamomilla*) exclusively) (Figure 3a). Additionally, one to three people mainly reported specific uses.

**Figure 4:**
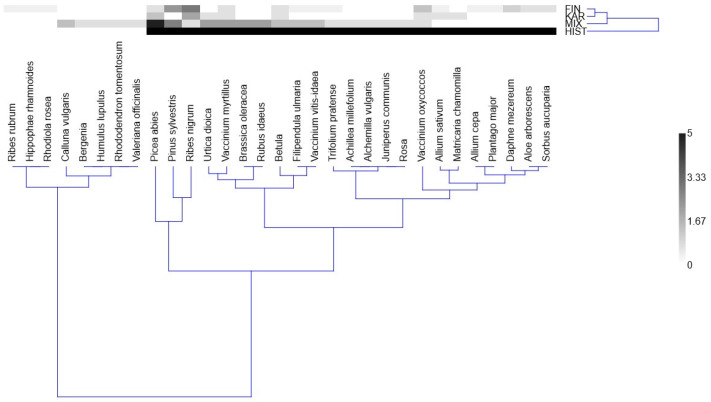
Comparison of the currently used medicinal plants in all groups with the historical (HIST) uses that overlapped with any of the current uses. A major part of the currently used taxa was also used according to historical sources. See Section 4.5 on how the values were calculated.

Figure 4 demonstrates that the current ethnomedicinal knowledge of ethnic Finns (FIN) is similar to that of ethnic Karelians (KAR). In contrast, the uses named by the group with mixed ethnic backgrounds (MIX) draw from both and are more similar to historical ethnomedicine (HIST). Of the 31 currently used taxa, 23 have also been used historically (out of 104 taxa, according to Kolosova et al. [22]). While the Karelians did not introduce any new taxa, the MIX group, despite being smaller, experimented with five new taxa. Notably, all 12 taxa reported as being used continuously have also been recorded in historical sources. The dendrogram of the species highlights species linked to specific groups and/or historical use. For example, *Hippophea rhamnoides*, *Ribes rubrum*, and *Rhodiola rosea* are absent from historical sources and only used by a few Finns. At the same time, *Juniperus communis*, *Achillea millefolium*, *Alchemilla vulgaris*, and *Rosa* were used by the MIX group only, although they were also present in historical sources. On a large scale, we can differentiate the cluster of plants mentioned in historical sources from those absent (divided only between the FIN and MIX groups).

## 3. Discussion

The number of documented medicinal plants in the current study (43 taxa mentioned by the 67 interviewees, of which 31 currently used) is much smaller than that in other European studies with similar samples sizes and is comparable only with the results obtained on the small island of Kihnu (Estonia), where only 23 persons were interviewed. In that study, the respective numbers were 33 (medicinal taxa mentioned) and 29 (currently used) [9]. In neighbouring Estonia, the use of 86 taxa was recorded in a sample of the same size, with 69 still in use in Seto and Võromaa [8]. Meanwhile, in the cross-border Seto region in the Russian Federation, 62 people reported the current medicinal use of 112 plant taxa [26], while in Latgale, Latvia, the use of 116 plant taxa (108 of these currently used) was recorded in 73 interviews [2].

The cross-ethnic Jaccard Index calculated in our study (around 0.45 for all uses and around 0.3 for current uses) is much smaller compared to 0.6 between Seto and Russians in Russia [26] and 0.55 between Seto and Võro in Estonia [8]. This suggests a higher ethnomedical diversity in the current study.

We observed that a high proportion of the medicinal plants currently in use in Finnish Karelia were also used in historical Karelian folk medicine [22]. This suggests that much of the local knowledge about medicinal plants in Finnish Karelia has deep historical roots, and it has been at least partially preserved despite modernisation. However, all the current uses are highly fragmented (with little consensus), with only a minimal number of plants being used by more than one person or in more than one way. A significant portion (63%) of all presentday uses has been recently acquired. This may indicate that the overlap between historical and current uses is due to the incorporation of newly acquired uses through books, mass media, and other sources. In general, the low number of uses does not support a viable ethnomedical system.

Ethnic Karelians have preserved their ethnomedicinal practices without incorporating those of the Finns, as evidenced by the absence of new taxa in their current usage repertoire compared to historical data. This contrasts with the high degree of integration observed in other communities, such as Buryat in Siberia, who have adopted elements of Russian herbal medicine alongside their traditional Tibeto-Mongolian healing practices [27]. Rather than being static or isolated, Tibetan medicine in Buryatia was shaped by external forces, including global medical trends and state healthcare systems. This integration reflects a broad process of knowledge exchange and adaptation, where local traditions blend with external therapeutic approaches to create a unique, hybrid medical ecology [28].

It could be suggested that the limited current use of medicinal plants among Karelians may be influenced by factors like forced relocation, loss of traditional lands, and the need to adapt to new, albeit similar, environments—phenomena evident in many displaced communities, for instance, the Yaghnobi in Tajikistan [29] or Sámi [30,31,32]. The data from ethnic Karelians may also reflect the fact that a significant portion of the interviewees from families with exclusively Karelian roots belong to the older generation. The oldest interviewees moved to Finnish Karelia as adults and had already established their plant use habits before the relocation. It was relatively easy to maintain their original traditions due to the similarity of the natural environment and because they moved short distances from their original homes.

Another possible reason for Karelian migrants and their descendants in Finland not adopting other ethnobotanical medicinal traditions is the significant post-war rise in the accessibility and quality of national healthcare in Finland. People of Karelian and Finnish origin reported that, previously, especially in small settlements, official healthcare was often unavailable, and people had to resort to traditional medicine. In the 1930s–1940s, traditional healthcare was predominantly used in Finnish Karelia. By the 1940s–1950s, traditional and official medicine were being used side by side. Starting in the 1960s, official healthcare became widely accessible, and traditional medicine began to be oppressed as non-scientific. The case of the local healer Anni Pölönen (1889–1960) illustrates this development. According to her grandson, whom we interviewed, and the works of her great-granddaughter [33,34], local doctors had initially been supportive of Anni’s work. However, after World War II (WWII), the legislation changed, and in 1956, Anni was tried for quackery and fined [33]. Her son was unable to continue her work, and after Finland entered the EU in 1995, herbal medicinal products became heavily regulated, with the sale of ingredients for self-made medicines prohibited [35].

For these reasons, the Karelian migrants who may have lost some of their original medicinal practices due to relocation in the 1940s did not have any a particular practical need to adopt local medicinal ethnobotanical practices. The similar nature of their new habitat, combined with the rise in official healthcare and a lack of support for traditional medicine by the receiving society, meant that there was little stimulus to integrate new practices. The prioritisation of modern state-sponsored healthcare systems over indigenous and local medicinal practices prevented their integration into broader healthcare frameworks. A similar process has been observed among the Sámi and Greenlandic Inuit [36,37,38,39].

Since the 1990s, however, the situation in Finland has partially changed. For example, cultural revitalisation programs in the Sevettijärvi-Näätämö community, a Skolt Sámi group in Northern Finland, have focused primarily on preserving and revitalising local food practices, while efforts to revive traditional healing methods remain limited [39]. Among our interviewees from North Karelia, people continue to rely on official healthcare rather than on traditional practices. However, there has been a recent shift toward recognising the value of “organic” and “homemade” products, which are promoted in Finland under the label *luomu* (short for *luonnonmukainen*, “conforming with nature”, meaning “organic”). This trend has influenced the attitudes towards traditional herbal medicine and its acceptance within official healthcare. Some interviewees mentioned that doctors nowadays occasionally recommend herbs or even use them themselves, and medicinal herbs are sold in the local natural product (*luontaistuotteet*) shops, such as in Nurmes. Overall, there is a growing trend to promote a holistic attitude toward health which also includes the use of medicinal herbs [40]. Those who want to learn the medicinal plant use themselves may avail of numerous books including those by the pioneer Toivo Rautavaara and, later, herbalists like Sinikka Piippo and Virpi Raipala-Cormier. Rautavaara (1905–1987), who was born in Karelia, introduced many of the modern types of natural products in Finland already in his first ground-breaking and highly popular herbal publication in 1942 [41], with more than a dozen reprints over several decades.

However, the presence of such publications, the organic product shops and a large number of plant-based medicines in the pharmaceutical system, has not yet resulted in widespread public recommendations of plants by medical professionals in Finland. While intense popularisation of herbal ethnomedicinal knowledge may have contributed to the current uptake of new and old plants, Finnish folk medicine remains highly literacy-based [23]. However, this trend has not entirely neutralised the negative attitudes that had previously prevailed in official healthcare. Despite recent introductions of new uses within the healthy lifestyle movement, the knowledge of medicinal plants remains highly eroded and fragile [42], with less than 25% of historically used plants still in practice.

In Finland, medicinal plants remain largely categorised under alternative medicine, and their use has significantly declined over the past decade, despite extensive promotion [43]. Finland boasts a highly accessible and robust healthcare system. However, according to the Finnish Institute of Health and Welfare [44], plants are only mentioned in the context of healthy food habits or as narcotic substances. Alternative medicine is often associated with the so-called “faith healing”, and plants are occasionally mentioned as part of this domain [45].

Under these circumstances, the practice of collecting plants for minor health issues and general well-being remains an important skill. It could help strengthen the connection between people and nature (e.g., [1]). Furthermore, the continuity of the practice encourages people to spend more time in nature, observe the rhythms, and acquire the “dark” (unacknowledged by public and unknown to science) part of the local knowledge [46]. Therefore, we suggest that more effort should be directed towards recognising the value of local plants in published herbals, aiming to reintroduce plant uses with historical local roots, provided their safety is confirmed.

## 4. Data and Methods

### 4.1. Study Region

Karelia has been a border area since the Middle Ages. The region of the ancient Finno-Ugric Korela groups was officially divided into eastern and western parts after the Peace Treaty of Nöteborg between Sweden and the Novgorod Republic in 1323 [47]. Over time, the divided region experienced cultural, linguistic, economic, social, and religious influences from the West (Sweden, Catholicism, and, later, Lutheranism from the 1500s) and the East (Novgorod, Principality of Moscow, and, later, Russia, with its Orthodox Church) [48]. The Karelian borderlands on both sides of the contemporary Finnish–Russian border have a history of shifting boundaries, largely due to wars [49]. These frequent changes have imbued the region with significant political and cultural complexity, fostering the mixing of languages and cultures. They have also created regional differences with the Karelian Isthmus, Ladoga Karelia, Olonets Karelia, and White Sea Karelia, all now in Russia, and North and South Karelia of Finland.

From 1809 until the end of WWII, much of Karelia was part of the Grand Duchy of Finland, which was an autonomous part of the Russian Empire until 1917. Following Finland’s independence, Eastern, or “Russian”, Karelia remained part of the Soviet Union, and in 1923, it was formally designated as the Karelian Autonomous Soviet Socialist Republic [50]. By the mid-1920s, the region was expanded to include areas with a larger Russian population, which led to a decrease in the proportion of Karelians. Industrialisation also contributed to an influx of settlers. Additionally, during the late 1930s, the establishment of concentration camps covered nearly one-third of the territory [51].

At the end of the nearly three-month Winter War (1939–1940), following the Soviet invasion of Finland, the USSR was forced to sign the Moskva Peace Treaty in 1940. However, as part of the agreement, a significant portion of Finnish Karelia was ceded to the Soviet Union, and approximately 420,000 refugees fled to Finland [52]. During the Continuation War (1941–1944), Finnish forces briefly regained the territory but were eventually forced to withdraw, and the local population was evacuated to other regions of Finland, including Finland’s North Karelia. The Soviet Union then relocated people from Russia, Belarus, Ukraine, and other regions to settle the area [53,54]. Only North and South Karelia remained within Finland’s borders.

The dissolution of the Soviet Union in 1991 resulted in a more open border, Finland’s accession to the European Union (EU), and reforms in regional governance that had a significant impact on local mobility. These changes led to increased economic and cultural ties between Finnish and Russian Karelia in the 1990s [53,55]. However, during the 2000s, especially in the past decade, contacts between the two regions have once again decreased due to the political situation in Russia.

Karelian is an endangered Uralic language, belonging to the Northern Finno-Ugric languages, together with Finnish [56]. In Finland, Karelians are bilingual, with approximately 11,000 fluent Karelian speakers and 30,000 with some knowledge, while others speaking only Finnish. In comparison, in Russian Karelia, where there are an estimated 9000 Karelian speakers (14,000 with some knowledge), all speakers also know Russian [57]. By the 1990s, anthropological fieldwork carried out by Estonians in Russian Karelia revealed that Karelian had largely disappeared as the primary language of communication in the region, being replaced by Russian. Today, the Karelian language has become strongly Russified [58].

### 4.2. Field Study and Data Collection

The province of North Karelia located in Eastern Finland, with its capital in Joensuu, covers approximately 23,000 km^2^, and is home to around 162,000 people (as of 2021) [59]. As Finland’s easternmost region, North Karelia shares a border of approximately 300 km with Russia. The climate is characterised by four distinct seasons, with cool continental summers and harsh, snowy, sub-Arctic winters. Precipitation occurs on about 30% of the days throughout the year, and the average annual temperature is around 5 °C. The region’s landscape is dominated by state-owned boreal forests, covering two-thirds of the area. These forests mainly consist of Norway spruce, Scots pine, and broad-leaved trees such as birch. Timber production is one of the region’s key industries, alongside forest and nature recreation and tourism [60].

**Figure 5:**
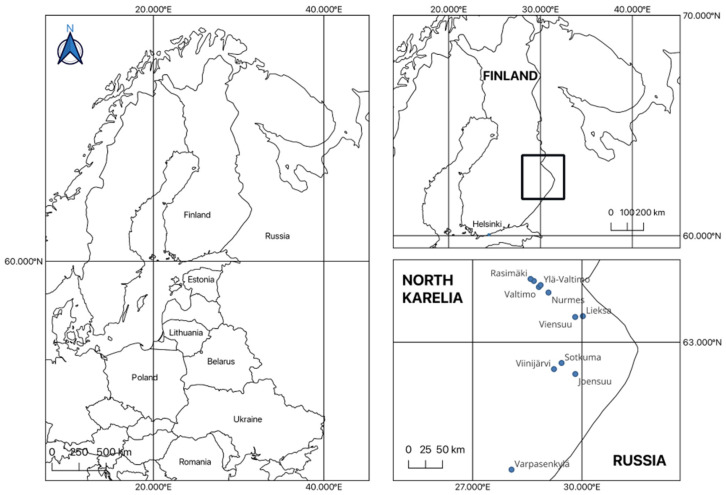
A map of the study area (black square). Designed in QGIS 3.22.

Fieldwork for the study was conducted from May to September 2018. The research primarily focused on the province of North Karelia, covering municipalities such as Joensuu, Lieksa, Nurmes, Valtimo, Ylä-Valtimo, Puukari, Rasimäki, Varpasenkylä, Viensuu, Viinijärvi, and Sotkuma, with additional interviews conducted in Helsinki, the capital of Finland (Figure 5). A total of 67 individuals were interviewed, including 34 of Karelian Finnish origin (FIN), 17 relocated Karelians and their descendants (KAR), and 16 individuals from mixed families (one parent Finnish, the other Karelian) (MIX).

The oldest interviewee was born in 1925, while the youngest was born in 1994, with an average age of 70. Of the 67 interviewees, 41 were women, and 26 were men. Nineteen study participants had higher education, twenty-nine had secondary education, seventeen had primary education, and two had no formal education. Most of the interviewees lived in rural areas, with only ten participants residing in larger urban centres such as Helsinki or Joensuu, and most were retired. Participants were identified and recruited through social networks, using snowball sampling, as random sampling was challenging due to the local cultural context. The sample size was deemed sufficient, with saturation reached after approximately ten interviews per group; after which, few new data points were introduced by additional interviewees.

The research protocol was approved by the Ethics Committee of Ca’ Foscari University of Venice. Prior to each interview, we provided a detailed description of the study and obtained informed consent from the participants, in accordance with the Code of Ethics of the International Society of Ethnobiology [61]. Upon obtaining consent, the interview began with questions about the use of wild food plants (see [1]), followed by inquiries related to ethnomedicine, including the use of medicinal plants. The initial “free listing” was typically brief. Participants were then asked about plant remedies for various diseases, beginning with ailments related to the head (e.g., headaches, colds, ear, and eye conditions, and sore throat), followed by internal organ diseases (e.g., stomach, heart, lung, and kidney issues), systemic disorders (e.g., joint diseases, diabetes, allergies, cancer, and immune system disorders), skin-related conditions (e.g., cuts, wounds, furuncles, and rashes), culture-bound diseases (e.g., the evil eye and nightmares), and other illnesses not yet mentioned. If any remedies from the free listing were not initially identified, participants were prompted to recall them. Interviewees were also asked to specify the first and last times they used each plant as accurately as possible. The ethnomedicine section concluded with questions on ethnoveterinary medicine, as detailed by Mattalia et al. [62]. The medicinal use of fungi in the region was addressed by Prakofjewa et al. [63]. For the purposes of this study, we considered plants and plant-based substances used for self-medication. The plants discussed were wild, cultivated, or purchased, and all parts and ingredients were identified to at least the genus level. We also inquired about specific methods of plant preparation.

The average duration of the interviews was two hours, and in some cases, the interviewees participated in small groups of two to three people. Following the interview, whenever possible, participants took a walk through their garden, around their homes, and the surrounding forest, during which additional plants or their uses were sometimes mentioned. Interviewees were encouraged to show all the plants they discussed whenever feasible. We also collected samples of dried plants available at the interviewees’ homes. These voucher specimens are stored in the herbarium of the Ca’ Foscari University of Venice (UVV), bearing numbers KAR02–KAR012 for herbarium specimens and KARDR01–KARDR24 for dried plant samples.

Some plants that were no longer used or were unavailable were identified by local names and descriptions provided by interviewees. Specific taxa, such as *Rosa*, *Betula*, *Trifolium*, and *Bergenia*, were identified solely at the genus level, as interviewees did not distinguish between species or the use of the plant was too distant in memory to confirm the species (e.g., *Dryopteris*). Botanical taxa nomenclature followed the Plants of the World Online [64].

Most of the interviews were recorded with the interviewee’s written consent and transcribed in Finnish (and Karelian for plant names). In one case, the recording was refused, and in another, the recording did not succeed, and we only took notes. Later, the information was tabulated in Microsoft Excel in English. The file was structured as Detailed Use Reports (DURs) of plant-based remedies, where each row contained the interviewee code, the scientific name of the plant, its local name, the part used, when it was used, the mode of preparation, its medicinal use, an original citation from the interview, and any specific comments, respectively.

### 4.3. Data Analysis

Descriptive statistics were performed in Microsoft Excel and Past 4.16c, where a *t*-test was performed to compare the influence of gender, age group, and belonging to a specific ethnic group on the number of plants used [65]. Venn diagrams were created using the tools by the Bioinformatics and Evolutionary Genomics laboratory at the University of Ghent [66] and Multiple List Comparator [67]. Proportional diagrams were created using the PAST Toolkit Venn diagram plotter software program [68]. Simple diagrams were plotted in Microsoft Excel, while RawGraph [69] was used for more complex diagrams.

The Jaccard Similarity Index (JI) was calculated as follows: (C/(A + B − C)), where C is the number of uses common to A and B, A is the number of uses in sample A, and B is the number of uses of sample B, following the methodology of González-Tejero et al. [70].

The Informant Consensus Factor (ICF) was calculated following Trotter and Logan [71] for etic disease categories, as follows: number of DUR minus the number of species used divided by the number of DUR minus one.

Following the temporal knowledge circulation framework [72], plant usage was categorised into three main groups to capture changes over time. These categories were based on the periods during which the plants were used:

- **Past uses** (no longer in active use)
- **Current uses:**
  ○ *Continuously used*—practices that have remained consistent over time (used more or less continuously from childhood to the present).
  ○ *Recently acquired uses*—practices adopted within recent years (approximately since 2000, with a focus on the last five years).

### 4.4. Disease Categorisation

Emic (local) disease names were translated into the etic (scientific) disease categories (Table 3) according to the International Classification of Primary Care, 2nd edition (ICPC-2, Updated March 2003) [73].

### 4.5. Comparison with Historical Sources

To compare current uses with historical records [22], hierarchical clustering was performed by applying a two-way Ward’s algorithm in Past 4.16c [65].

The colour gradient scale was calculated by dividing the number of DUR for each plant by the number of interviewees in each group, then multiplying the result by ten. The threshold for all historical uses was set at five, slightly above the highest gradient of uses to be differentiated in the illustration. Kolosova et al. [22] refers to historical use, the most recent one being from the 1950–1960s. It also covers only the Russian part of Karelia.

## 5. Conclusions

In Finnish North Karelia, we documented a limited repertoire of medicinal plants, most of which have remained in the past. While some differences in plant usage were observed between local Finns and Karelian migrants for the past, the remaining scattered knowledge does not provide sufficient grounds to draw definitive conclusions about broader trends or trajectories in local medicinal knowledge.

The most significant finding relates to the role of the state and its regulations in either supporting or oppressing the use of plants. The lack of state support for traditional plant use in post-war Finland, coupled with improvements in the quality and accessibility of official medicine, contributed to the marginalisation of plant-based ethnomedicine. Over time, the formal classification of plant uses as “alternative medicine” further diminished the practice. This has led to the erosion of the historical plant-based ethnomedicinal system and local knowledge.

Moving forward, greater efforts should be made to acknowledge and support the safe traditional uses of local plants while also promoting healthy wild food foraging. Such initiatives could help foster sustainable human–nature interactions in a bioculturally responsible manner. The current trend in Finland towards a more holistic approach to healthcare offers some hope in this regard, although resistance from official medical practitioners remains considerable.

## Figures and Tables

**Table 1:** Plants used in North Karelia, Finland, currently and in the past.

Scientific Name (Family), Herbarium №, W(ild) or (C)ultivated	Local Name (s)	Plant Part	Etic Disease Category	Past DUR	Current DUR
*Allium cepa* L. (Amaryllidaceae), C, *	sipuli (Fin)	Bulb	Ear	1	
			Gen	1	1
			Resp	2	
*Allium sativum* L. (Amaryllidaceae), C	valkosipuli (Fin)	Bulb	Ear	2	
			Gen		1
			Resp	1	
*Aloe arborescens* Mill. (Asphodelaceae), C	aloe (vera) (Fin)	Leaves	Skin		2
*Achillea millefolium* L. (Asteraceae), KARDR15, KARDR22, KAR06, W, *	siankärsämö (Fin)	Flowers	Dige		1
		Leaves	Skin *	1	
*Matricaria chamomilla* L. (Asteraceae), C, *	kamomilla (Fin)	Aerial parts	Skin		1
		Leaves	Psych	1	
*Pseudopodospermum hispanicum* (L.) Zaika, Sukhor. & N.Kilian (Asteraceae), C	mustajuuri (Fin)	Roots	Gen	1	
*Taraxacum officinale* F.H.Wigg. s.l. (Asteraceae), KAR16, KARDR25, W, *	voikukka (Fin)	Roots	Dige	1	
			Gen	1	
		Sap	Skin *	2	
*Betula* sp. (Betulaceae), KARDR28c, KARDR10, KAR12, W, *	rauduskoivu (Fin), koivu (Fin)	Leaves	Endocr		1
			Skin *	1	
			Uro	1	
		Sap	Gen	1	3
		Charcoal	Dige	2	
			Gen	1	
		Twigs	Blood		1
			Musc	3	
		Birchbark	Skin	1	
*Armoracia rusticana* G.Gaertn., B.Mey. & Scherb. (Brassicaceae), C	piparjuuri (Fin)	Roots	Resp	1	
*Brassica oleracea* L. (Brassicaceae), C, *	kaali (Fin), keräkaali (Fin)	Leaves	Dige		1
			Endocr	1	1
			Musc	1	
			Skin	2	1
*Humulus lupulus* L. (Cannabaceae), C	humala (Fin)	Cones	Psych		1
*Valeriana officinalis* L. (Caprifoliaceae), KARDR16, C/W	valeria (Fin)	Flowers	Psych		1
*Rhodiola rosea* L. (Crassulaceae), C	ruusujuuri (Fin)	Roots	Gen		1
*Juniperus communis* L. (Cupressaceae), W, *	kataja (Fin)	Fruits	Gen	1	
			Uro	3	
		Twigs	Gen		1
*Hippophae rhamnoides* L. (Elaeagnaceae), C	tyrni (Fin)	Fruits	Dige		1
			Gen		1
*Calluna vulgaris* (L.) Hull (Ericaceae), KARDR07, KAR36, W	kanerva (Fin)	Flowers	Psych		2
			Uro	1	
*Rhododendron tomentosum* Harmaja (Ericaceae), KARDR26, W	suopursu (Fin)	Aerial parts	Endocr		1
			Gen	1	
			Musc	1	
*Vaccinium myrtillus* L. (Ericaceae), KAR03, W, *	mustikka (Fin), musčoi (Kar), mustoi (Kar), must’oi (Kar)	Fruits	Dige *	17	2
			Eye	1	1
			Gen	5	2
			Musc	1	
			Resp	5	
		Leaves	Gen	1	1
*Vaccinium oxycoccos* L. (Ericaceae), W, *	karpalo (Fin), garbalo (Kar), karbalo (Kar)	Fruits	Gen	1	
			Men		1
			Resp	4	1
			Uro	7	4
*Vaccinium vitis-idaea* L. (Ericaceae), KAR04, KAR37, KARDR16, W, *	puolukka (Fin), buola (Kar), puoloi (Kar)	Fruits	Dige	1	
			Gen	3	1
			Resp *	3	
			Uro		2
*Trifolium pratense* L. (Fabaceae), KARDR11, KARDR08, KAR32, W	puna-apila (Fin)	Aerial parts	Wom		1
*Trifolium* sp. (Fabaceae), W, *	apila (Fin)	Flowers	Musc	1	
*Ribes nigrum* L. (Grossulariaceae), KAR11, C, *	mustaherukka (Fin), mustaviinimarja (Fin), karpalo (Fin)	Fruits	Dige	1	
			Gen	9	5
			Resp	21	7
		Leaves	Gen	2	
*Ribes rubrum* L. (Grossulariaceae), C	punaherukka (Fin),	Fruits	Resp	1	1
*Ribes spicatum* E.Robson (Grossulariaceae), W	punaherukka (Fin)	Fruits	Resp	2	
*Mentha × piperita* L. (Lamiaceae), C, *	piparminttu (Fin)	Aerial parts	Resp	1	
*Linum usitatissimum* L. (Linaceae), C	pellava (Fin)	Seeds	Dige	1	
*Picea abies* (L.) H.Karst. (Pinaceae), KAR06a, W, *	kuusi (Fin)	Resin	* Skin	9	6
		Twigs (young)	Gen	2	
			Musc	1	
		Shoots and young twigs	Resp	1	4
*Pinus sylvestris* L. (Pinaceae), KARDR24, W, *	mänty (Fin), petäjä (Kar)	Resin	Blood	1	
			Dige	2	
			Gen	1	
			Resp	1	
			Skin *	10	
		Roots	Resp	1	3
		Tar	Endocr		1
			Gen		3
			Resp		1
			Skin	9	4
		Charcoal	Skin	1	
*Plantago major* L. (Plantaginaceae), KAR01, W, *	piharatamo (Fin), ratamo (Fin)	Leaves	Musc	1	
			Skin *	30	1
*Avena sativa* L. (Poaceae), C	kaura (Fin)	Seeds	Dige	1	
*Dryopteris* sp. (Polypodiaceae), W	alvejuuri (Fin)	Roots	Dige	1	
*Alchemilla vulgaris* L. (Rosaceae); KARDR29, W, *	poimu (Fin)	Leaves	Dige *		1
*Filipendula ulmaria* (L.) Maxim. (Rosaceae), KARDR09, KARDR12, KARDR28d, KAR13, W, *	mesiangervo (Fin)	Flowers/Leaves	Gen		2
			Resp		1
*Rosa* sp. (Rosaceae), KAR17, W, *	ruusu (Fin)	Fruits	Gen *	3	1
*Rubus arcticus* L. (Rosaceae), KARDR14, KARDR23, KAR23, W	mesimarja (Fin) orhoi (Kar)	Fruits, sometimes leaves	Resp	2	
*Rubus chamaemorus* L. (Rosaceae), W, *	lakka (Fin)	Fruits	Resp *	3	
*Rubus idaeus* L. (Rosaceae), KARDR28b, KARDR06, KAR18, W, *	vadelma (Fin), vattu (Fin), vagoi (Kar)	Fruits	Resp *	4	
			Wom		1
		Leaves	Dige	1	
			Gen	1	2
			Resp	2	
*Sorbus aucuparia* L. (Rosaceae), KAR19, W, *	pihlaja (Fin)	Fruits and leaves	Gen		2
*Bergenia* sp. (Saxifragaceae), C	vuorenkilpi (Fin)	Aerial parts	Blood		1
*Nicotiana rustica* L. (Solanaceae), C, *	tupakka (Fin)	Leaves	Dige	2	
			Ear	11	
*Daphne mezereum* L. (Thymelaeaceae), W, *	näsiä (Fin), lehtonäsiä, riianmarja (Fin)	Fruits	Dige *	1	
			Gen *	2	1
			Musc	2	1
			Skin		1
*Urtica dioica* L. (Urticaceae), KARDR13, KAR02, W, *	nokkonen (Fin), čiiloiheinä (Kar), vihulainen (Kar)	Aerial parts	Gen		3
			Resp		1
			Skin *		1

*—Plant/use recorded in historical written sources. Fin—Finnish, Kar—Karelian. Blood—Blood, Blood-Forming Organs, and Immune Mechanism; Dige—Digestive; Ear—Ear; Endocr—Endocrine/Metabolic and Nutritional; Eye—Eye; Gen—General and Unspecified; Men—Male Genital; Musc—Musculoskeletal; Psych—Psychological; Resp—Respiratory; Skin—Skin; Uro—Urological; Wom—Female Genital diseases; DUR—Detailed Use Report.

**Table 2:** Comparison of plants mentioned per person and the sum of the Detailed Use Reports (DURs) per person across different groups of interviewees. With bold are highlighted more knowledgeable groups. **—*p* < 0.05, ***—*p* < 0.001.

		Plants	DUR	
Ethnic group	Fin	3.27	4.24	*n* = 31
**	Kar	3.36	4.71	*n* = 14
	**Mix**	**5.28**	**9.31**	*n* = 13
Gender	Male	3.05	4.38	*n* = 21
***	**Female**	**4.22**	**6.19**	*n* = 37
Age	Over 70	3.31	4.44	*n* = 32
**	**55–70**	**4.95**	**7.84**	*n* = 19
	Up to 55	2.88	4.29	*n* = 7
	All	3.79	5.53	*n* = 58

**Table 3:** Correspondence between emic disease names with etic disease categories and their frequency of mentioning.

Application	DUR
**(Blood) diseases**	3
decreasing blood pressure	1
good for blood circulation	1
varicose veins	1
**(Dige)stive tract diseases**	36
Cancer	2
Diarrhoea	10
Heartburn	1
intestinal problems	2
intestinal worms	1
liver problems	1
stomach problems	13
Stomach ache	1
Toothache	5
**(Ear) diseases**	14
ear infections	1
Earache	13
**(Endocr)inological diseases**	5
Gout	1
Metabolism	1
Struma	1
Swelling	2
**(Eye) diseases**	2
good for sight	1
helps for night vision	1
**(Gen)eral diseases**	69
Antioxidant	1
body cleansing	2
body detoxication	1
Cancer	1
children’s diseases	1
Cold	1
Fever	14
Hangover	2
Headache	1
Healthy	19
Inflammations	4
Medicine	4
Panacea	6
preventing cancer	1
Prophylactics	1
Rickets	2
Tuberculosis	1
vitamin C	5
whooping cough	1
**(Men) diseases**	1
prostate problems	1
**(Musc)ulo skeletal diseases**	12
back problems	2
for pains and tired feet	1
joint rheumatism	1
joints and ache	1
muscle strains	1
Muscles	1
Rheumatism	2
rheumatoid arthritis	1
sore back	1
sore limbs	1
**(Psych)ological diseases**	5
calming down	1
calming down/sleep	1
for sleeping	3
Stress	1
**(Resp)iratory diseases**	74
Angina	1
Bronchitis	1
Cold	33
Cough	10
Flu	19
nasal catarrh	1
nose constipation	1
sore throat	7
when losing voice	1
**(Skin) diseases**	81
Abscesses	2
Allergy	3
bite of wasp	1
Bites	1
Bruises	2
Burns	3
Eczemas	1
Itching	1
nettle sting	1
Pimples	2
Rash	2
Scratch	1
Skin	2
skin (all body)	1
skin problems	4
to brighten the skin	1
Warts	5
Wounds	48
**(Uro)logical diseases**	18
Cancer	1
cancer (for women)	1
diuretic/fluid balance	1
kidney problems	1
urinary infection	8
urinary problems	2
urinary problems in men	1
urinary tract infection	3
**(Wom)en diseases**	2
women diseases	1
women problems	1

## Data Availability

Data are available upon request to the corresponding authors.

## Abbreviations

W—wild; C—cultivated; ICF—Informant Consensus Factor; UR—Use Report; JI—Jaccard Index; KAR—Karelians; FIN—Fins; MIX—mixed; DUR—Detailed Use Report; ICPC-2—International Classification of Primary Care; EU—European Union; WWII—World War II; USSR—The Union of Soviet Socialist Republics.
